# Ganglioside Biochemistry

**DOI:** 10.5402/2012/506160

**Published:** 2012-12-19

**Authors:** Thomas Kolter

**Affiliations:** Program Unit Membrane Biology & Lipid Biochemistry, LiMES, University of Bonn, Gerhard-Domagk Straße 1, 53121 Bonn, Germany

## Abstract

Gangliosides are sialic acid-containing glycosphingolipids. They occur especially on the cellular surfaces of neuronal cells, where they form a complex pattern, but are also found in many other cell types. The paper provides a general overview on their structures, occurrence, and metabolism. Key functional, biochemical, and pathobiochemical aspects are summarized.

## 1. Introduction

**Figure 1:**
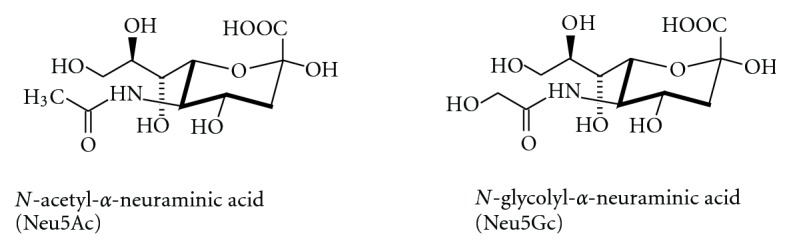
Sialic acids.

Together with glycoproteins and glycosaminoglycans, glycosphingolipids (GSLs) contribute to the glycocalyx that covers eukaryotic cell surfaces. Gangliosides are sialic acid-containing glycosphingolipids and provide a significant part of cell surface glycans on neuronal cells. GSLs are lipids that contain a sphingoid base and one or more sugar residues [1]. Sialic acids (Figure 1) are nine-carbon sugars biosynthetically formed from *N*-acetylmannosamine and phosphoenolpyruvate [2, 3]. With a mean p*K*_*A*_ value of around 2.6, they are more acidic than the majority of carboxylic acids and negatively charged at most physiological pH values. The name “ganglioside” was coined by the German biochemist Klenk (1896–1971) and assigned to a group of acidic GSLs that he isolated from ganglion cells [4, 5] and from the brains of patients who suffered from the so-called amaurotic idiocy [6, 7]. Sialic acid was first isolated from submaxillary mucin in 1936 [8]. Its structure was elucidated in the nineteen fifties by different groups and it was found to be identical to that of the *N*-acetylneuraminic acid isolated by Klenk and Faillard. The first structure of a ganglioside was elucidated in 1963 by Kuhn and Wiegandt [9]. In 1962, Svennerholm suggested a nomenclature of brain gangliosides [10, 11]. The biochemical defects underlying the diseases formerly known as amaurotic idiocy, GM1-gangliosidosis [12], Tay-Sachs- [13], and Sandhoff disease [14] were identified by Sandhoff and others in the 1960s.

## 2. Structure and Nomenclature

In their structures, gangliosides combine a glycan and a lipid portion and contribute to both, the cellular lipidome and the glycome/sialome [15]. A great variety of carbohydrate sequences are found within the GSLs [16], including the gangliosides [17]. Although carbohydrate residues of different structure, linkage, and anomeric configuration occur in GSLs, only a limited number of the so-called series with characteristic carbohydrate sequences are found within evolutionary related organisms (Table 1). Within the gangliosides, sialic acids can be attached only to a few of the GSL series, in adult mammals especially to the ganglio series.

Among the sialic acids, *N*-acetylneuraminic acid is the most frequently found member in humans, but also *N*-glycolylneuraminic acid is abundant in many other species (Figure 1). A total of more than 50 different sialic acids have been described [18, 19]. They can be *O*-acetylated in positions 4, 7, or 9 [20], but also *N*-deacetylated, *O*-methylated, sulfated, or modified by lactonization [21] (see Figure 8).

**Figure 2:**
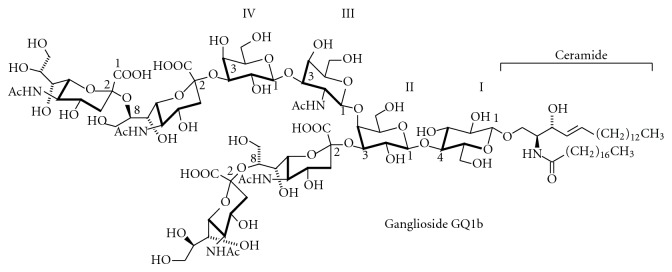
Structure of GQ1b, one of the most abundant gangliosides in adult human brain, which is involved in long term potentiation, synaptic plasticity, and improvement of cognitive function [23].

The nomenclature of GSLs specifies the glycan part of these lipids. Two ganglioside nomenclature systems are currently in use to assign names to the corresponding structures. Most researches prefer the short-hand nomenclature according to Svennerholm, which was initially based on the migration order of ganglio-series gangliosides in chromatography [10]. Later on, it has been extended to other root structures. The more comprehensive IUPAC system [22] is less frequently applied. According to Svennerholm, a core structure of neutral sugars define the name of a respective series, in which the pyranose forms of D-galactose (Gal), D-*N*-Acetyl-glucosamine (GlcNAc), or D-*N*-Acetylgalactosamine (GalNAc) are attached in defined order and linkage to lactosylceramide (Gal*β*1,4Glc*β*1Cer) or *β*-galactosylceramide (Gal*β*1Cer). The names contain information about the series (“G” = ganglio, “L” = lacto), the number of sialic acids (“A” = 0, “M” =1, “D” = 2, “T” = 3, “Q” = 4, “P” = 5, “H” = 6, “S” = 7), and, indirectly, on the number of uncharged carbohydrates: initially it has been assumed that this number cannot exceed 5, so that the name “ganglioside GM1” indicates that this ganglioside contains (5 − “1” = 4) neutral sugars of the ganglio series. This series is defined by the sequence Gal*β*1-3GalNAc*β*1-4Gal*β*1-4GlcCer. Sialic acids can be attached once, twice, or severalfold to different positions within the core structures. Most often, they are found in *α*2,3-linkage to the “inner” or “outer” galactosyl residue, and in *α*2,8-linkage to other sialic acids. Ganglioside GM1 bears one sialic acid moiety connected to the 3-OH-group of the galactosyl residue in position II of the gangliotetraose moiety (see also Figure 7). The corresponding IUPAC-IUBMB short-hand name is II^3^Neu5AcGg_4_Cer. Structures of ganglio-series gangliosides can also be derived from the scheme of ganglioside biosynthesis (see below; Figure 12). In general, ganglio-series GSLs of the 0-series bear no sialic acids on the galactose in position II, of the a-series bear one, of the b-series bear two, and of the c-series bear three sialic acid residues. However, GM1b and GD1c have a “b” and “c” in their names, although both are 0-series gangliosides (see the scheme of ganglioside biosynthesis, Figure 12). GM4 is a gala-series ganglioside, although the “G” suggests ganglio series.  Figure 2 shows the structure of ganglioside GQ1b, one of the most abundant gangliosides in adult human brain (G = ganglio series, Q = 4 sialic acids, 5 − 1 = 4 neutral carbohydrate residues, and b-series = 2 sialic acids attached to the “inner” galactose).

**Figure 3:**
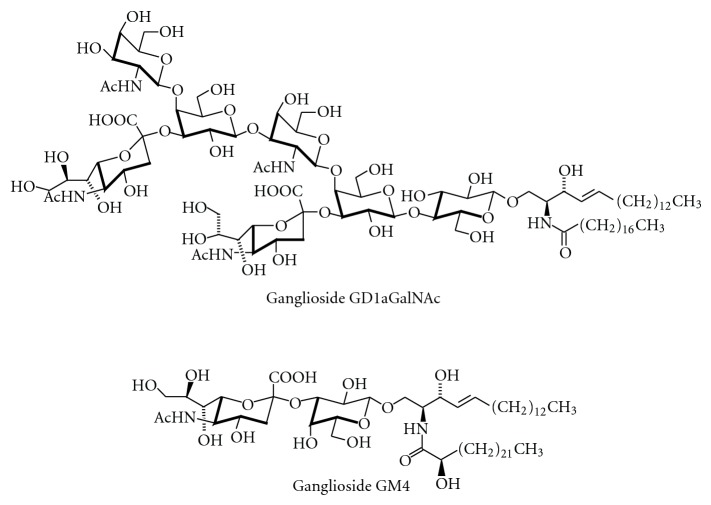
GD1aGalNAc and GM4.

Ganglioside core structures can be additionally modified; they can be elongated, such as in GD1aGalNAc [25] (Figure 3). This ganglioside occurs, for example, on spinal neurons [26, 27] and can give rise to autoantibodies as a cause of variant forms of the Guillain-Barré syndrome [28, 29] and other neuropathies [30]. A modified GM2 derivative that contains taurine in amide linkage to the sialic acid carboxyl group has been identified in the brain of patients with Tay-Sachs disease [31]. Hybrid-type GSLs and gangliosides with postglycosylation modifications add further complexity to this substance class [32]. As an example, lacto-ganglio hybrid-type gangliosides have been identified in bovine brain [33].

**Figure 4:**
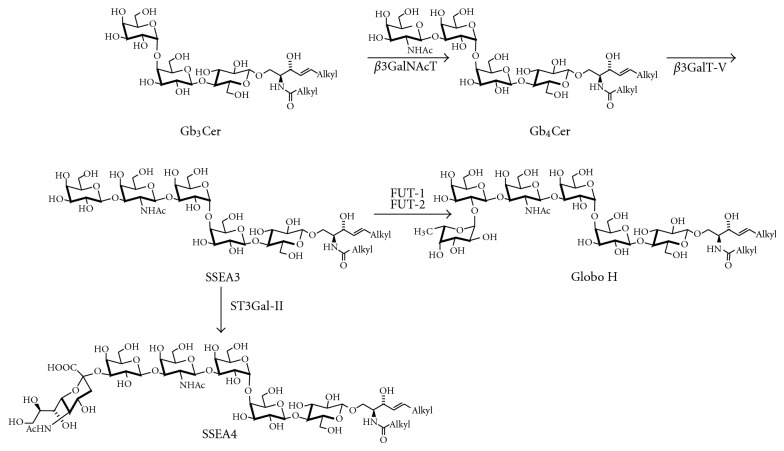
Formation of SSEA-4 from globotriaosylceramide (Gb3Cer). SSEA, stage-specific embryonic antigen; T, transferase; FUT, fucosyltransferase (modified from [24]).

Most gangliosides found in adult mammals belong to the ganglio, gala, lacto, and neolacto series. Ganglioside GM4 (Figure 3), a member of the gala series, has the structure NeuAc*α*2,3Gal*β*1Cer and is often found with an *α*-hydroxy-fatty acid within the ceramide moiety. During development, also gangliosides with other core structures are transiently formed, such as the stage-specific embryonic antigen SSEA-4, a ganglioside of the globo series [34] (Figure 4). In adults, globo-series gangliosides occur on human erythrocytes [35], in human kidney [36], and on various stem cells [37]. For example, SSEA-4, but not SSEA-3 or Globo-H (Figure 4), is expressed in cord blood-derived mesenchymal stem cells [24].

**Figure 5:**
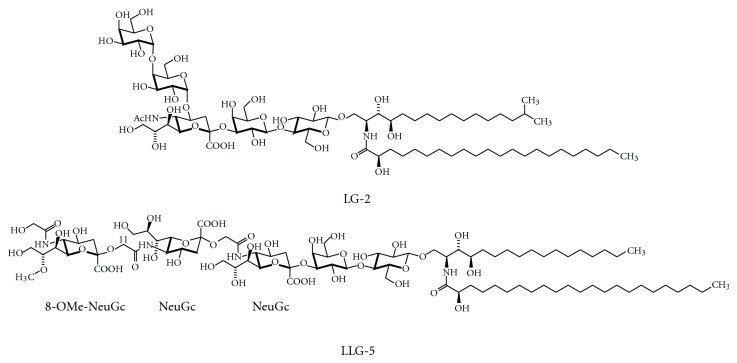
Examples for gangliosides from echinoderms: LG-2 from the starfish *Astropecten latespinosus* [47] and LLG-5 from the starfish *Linckia laevigata* [48–50].

With the exception of echinoderms (marine organisms of typically pentaradial symmetry), gangliosides are usually absent from invertebrates. Arthropods, for example, form acidic GSLs with a Man*β*1,4Glc*β*1,1′Cer core, which contain glucuronic acid instead of sialic acids. For gangliosides of echinoderms [38–42], there is no systematic short-hand nomenclature. They show structural features uncommon to mammalian gangliosides, such as sialic acid residues within the oligosaccharide moieties (e.g., LG-2, Figure 5), *α*2,11-linked sialic acids (e.g., LLG-5, Figure 5), sialic acid methylation or sulfation, or a glycosyl inositolphosphoceramide core, for example, [43, 44]. In cultured neurons, echinodermal gangliosides show neuritogenic and growth-inhibitory activities. In this regard, they are more potent than other gangliosides [45, 46] and potentiate the neuritogenic effect of nerve growth factor.

**Figure 6:**
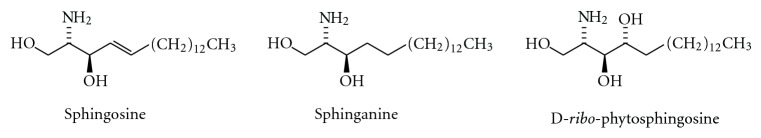
Representative structures of sphingoid bases, in this case (d18:1), (d18:0), and (t18:0).

Heterogeneity is not only found within the glycan part, but also within the ceramide moiety. This can consist of different sphingoid bases [51], sphinganine, sphingosine, and phytosphingosine of different chain lengths (Figure 6), which can be further modified by *O*-acetylation [52]. In higher animals, C_18_- and C_20_-sphingosine are the most abundant sphingoid bases of gangliosides. 

The fatty acids found in the ceramide part of gangliosides are mostly saturated. *α*-Hydroxylated fatty acids [53] are not frequently found in brain gangliosides, but are, for example, abundant in gangliosides from intestine, liver, or kidney, and in GM4. To specify the lipoform of a ganglioside, designations such as (d18:1/18:0)GM3 are used for a II^3^Neu5AcLacCer with a sphingosine (d = dihydroxy, 1 = one double bond; see also Figure 6) of 18 carbons and a stearoyl residue (18:0) within the ceramide portion. The functional consequences of the heterogeneities in the lipid component are largely unknown, but the lipid part can mask the receptor function of ganglioside glycans via interaction with membrane cholesterol [54, 55]. As another example, the ceramide portion of GM1 dictates retrograde transport of cholera toxin bound to GM1, and only GM1 with unsaturated acyl chains is sorted from the plasma membrane to the trans-Golgi network and the ER [56]. Ganglioside profiling with respect to glycan and ceramide structures is more and more in the focus of ganglioside analysis.

## 3. Occurrence

**Figure 7:**
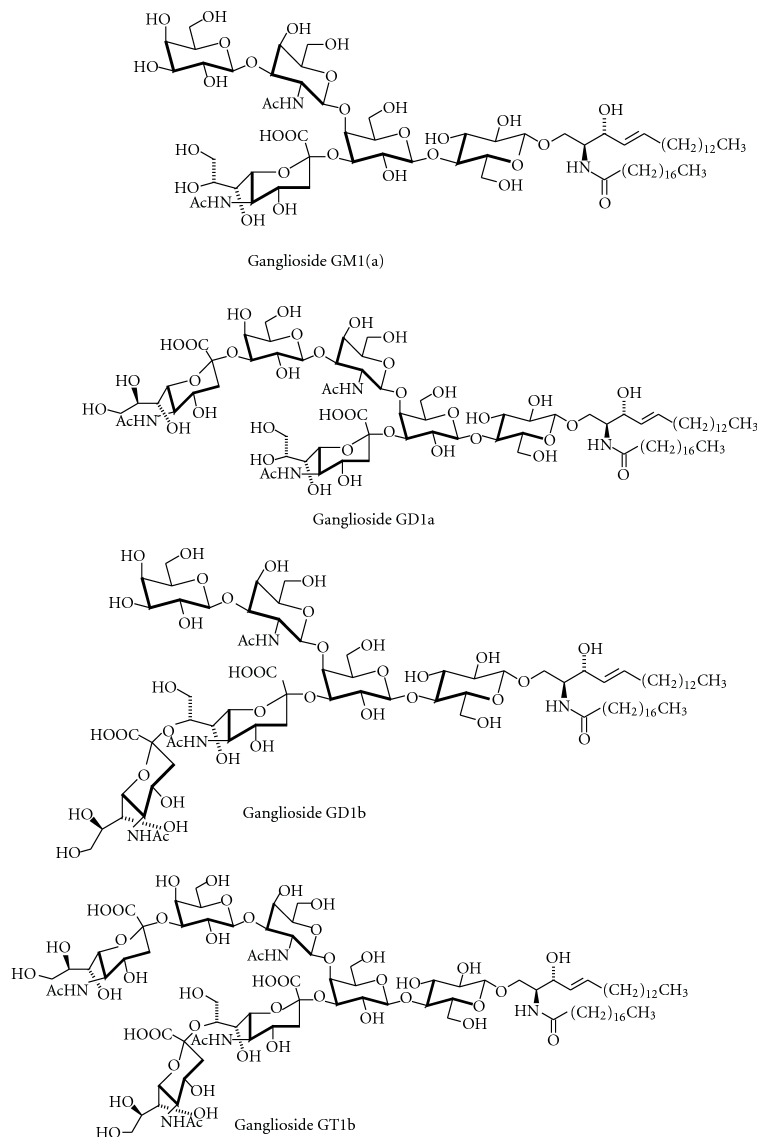
Representative structures of gangliosides that are abundant in adult human brain.

Gangliosides are especially abundant in the brain, where their occurrence in the grey matter is about 5-fold higher than in white matter. In adult human brain regions, the values range from 2 to 14 *μ*g lipid-bound sialic acid/mg protein [57]. In the brain, ganglioside expression correlates with neurogenesis, synaptogenesis, synaptic transmission, and cell proliferation [58, 59]. In cultured murine hippocampal neurons, axonogenesis, but not dendritogenesis, is accompanied by an increase in the formation of complex gangliosides and by a shift from the a- to b-series [60]. In extraneural tissues, the ganglioside content is one- to two- orders of magnitude lower than in the brain; relatively high concentrations of ganglio-series gangliosides are found in bone marrow, erythrocytes, intestine, liver, spleen, and testis, GM4 in kidney, and SSEA-4 in embryonic stem cells. Cellular gangliosides form in part complex, cell-type- and tissue-specific glycan patterns [61]. These are not stable with time, but change with physiological and pathophysiological processes such as cell growth, differentiation, viral transformation, ontogenesis, oncogenesis, embryogenesis [62, 63], lactation, or tumor progression [64]. Gangliosides of the ganglio-series are especially found in the nervous system, where they contribute to 10–12% of the lipid content [65]. During brain development, the ganglioside pattern changes from the prevalence of the simple gangliosides GM3 and GD3 to more complex ones such as GD1a and GT1b [66] (for structures, see Figures 7 and 11). Ganglioside content and composition of the brain change also during aging: for example, the amount of lipid-bound sialic acid decreased from 1070 *μ*g/g wet weight in a 25-year-old healthy proband to 380 *μ*g/g wet weight in a 85-year-old individual. Despite this, the concentrations of GQ1b, GT1b, and GD1b increase with age at the expense of GM1 and GD1a [67] (for structures, see Figures 2 and 7). Changes in ganglioside composition with age also occur in liver [68]. There are only indications on the functional consequences of such changes [23].

Gangliosides are also found in serum. There, especially GM3, GD3, GD1a, GM2, GT1b, sialylneolactotetraosylceramide (Figure 10), GD1b, and GQ1b are present, where about 98% of them are transported by serum lipoproteins, predominantly by LDL (66%), followed by HDL (25%) and VLDL (7%) [69]. After the discovery of extracellular microvesicles (formerly called microparticles) [70], which were not distinguished from lipoproteins in earlier experiments, it might turn out that these assignments have to be revised. 

Experiments in rats have shown that after injection of [^14^C]sialic acid-labeled gangliosides GM3 and [^3^H]sphingosine-containing labeled ganglioside GM1, the GM1 and GM3 probes had serum half-lives of 1.4 and 1.8 h, respectively. After three hours, 75% of the GM1 and 38% of the GM3 probes were taken up by the liver, and a smaller extent in the central nervous system, kidneys, and lung [71].

Subcellularly, the majority of gangliosides resides in the plasma membrane [72]. However, gangliosides also occur in organellar membranes such as in mitochondria, where GD3 regulates apoptosis [73], and in the nucleus, where they are involved in Ca^2+^ balance [74, 75].

The glycans found in gangliosides are sometimes modified by the acylation of sialic acid residues in different positions [20]. *O*-Acetylated sialic acids in gangliosides occur especially in growing cells and tissues and are regarded as oncofetal markers present on different tumors [76]. They also serve as receptors for Influenza C viruses or coronaviruses [77].

Another modified sialic acid is *N*-glycolylneuraminic acid (Neu5Gc) [42]. With the exception of certain tumors and in fetuses, it is found only in trace amounts in human tissues [78]. As a component of glycoconjugates, Neu5Gc is known as the Hanganutziu-Deicher antigen [79]. It is abundant in many species of the Deuterostome lineage, including simians, mice, rat, beef, pork, or lamb, but is nearly absent from birds and reptiles [80]. Neu5Gc on glycoconjugates contributes to xenoantigenicity in pig-human xenotransplantation [81], and in cats, Neu5Gc distinguishes the blood groups A and B: [Neu5Gc]_2_GD3 is found in feline blood group A erythrocytes, [Neu5Ac]_2_GD3 on blood group B, and feline blood group AB erythrocyte membranes contain [NeuGc]_2_GD3, [Neu5Ac,Neu5Gc]GD3, and [NeuAc]_2_GD3 [82].

Humans cannot synthesize Neu5Gc due to an irreversible inactivation of the CMAH gene on chromosome 6p21.32 encoding Cytidine monophosphate-*N*-acetyl-neuraminic acid hydroxylase [83]. This enzyme converts CMPNeu5Ac to CMPNeu5Gc and its function is thought to be lost during a “sialoquake” in human evolution [84, 85]. Determination of Neu5Gc and Neu5Ac-containing gangliosides is either achieved by classical chromatographic techniques combined with antibody staining [86], or, with higher sensitivity, by combination of chromatography with ESI-MS [87]. A potential application is the immunochemical detection of [Neu5Gc]GM3 as biomarker of nonsmall-cell lung cancer [88].

**Figure 8:**
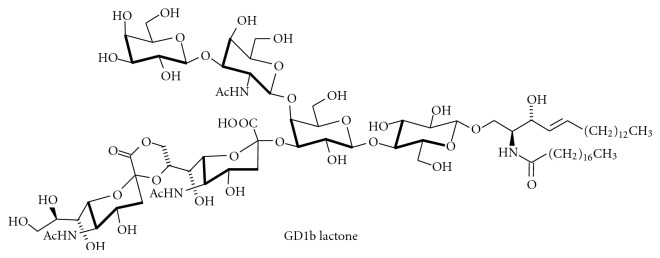
Example of a ganglioside lactone.

Also ganglioside lactones (Figure 8) have been detected in various tissues, for example, GD3 lactone in mouse brain [89] and GD1b lactone in human brain [90]. Ganglioside lactones are more immunogenic than gangliosides [91] and occur on tumor cells such as melanoma as tumor-associated antigens. *In vitro*, lactonization of gangliosides can be followed by a strong negative Cotton effect at 235 nm in CD spectroscopy [92].

Temporal and spatial differences are also observed for the ganglioside lipid part. In undifferentiated neuronal cell cultures, gangliosides with C_20_ sphingosine are present only in trace amounts, but their content increases with the onset of cell differentiation [93]. In rat brain, the fraction of gangliosides containing C_20_ sphingosine increases with age [94] in cerebellum [95] or forebrain [96]. Spatial differences regarding the sphingoid base chain length have also been detected in mice: while gangliosides containing C_18_ species were widely distributed throughout the frontal brain, C_20_ species are selectively localized along the entorhinal-hippocampus projections [97]. Fatty acid and sphingoid base composition is also different between human motor and sensory nerves [98].

**Nutrition**

Since gangliosides are components of most vertebrate cell types, they are ingested with the nutrition, for example, with egg yolk (GM3, GM4, and GD3), meat, or in milk [99]. Milk contains gangliosides, especially GD3 and GM3, in the membrane fraction of the fat globule. Dietary gangliosides modify the intestinal microflora and prevent infections during early infancy [100]. In infants, more than 80% of dietary gangliosides survive the passage through the stomach, in part with acid-catalyzed lactonization, and are absorbed in the intestine [99]. Ingestion of dietary gangliosides leads to an increase of gangliosides in serum. In human nutrition, sialic acid derived from gangliosides and other glycoconjugates is an essential nutrient for the rapidly growing brain in infants [59]. The pathophysiological consequences of nutritional Neu5Gc uptake are unknown.

## 4. Analysis

In the past, ganglioside structures and levels were obtained by comprehensive chemical analysis, while nowadays this is attempted within lipidomics using mass spectrometry as the key technology [101].

In general, gangliosides are isolated from tissues and body fluids by chloroform-methanol extraction [102, 103]. Extraction efficiency can increase when small amounts of water are present in the extraction solvent [104], for example, using the solvent system chloroform : methanol : water (5 : 5 : 1) [105]. When extraction is followed by a partition step such as that developed by Folch et al. [106], gangliosides—in contrast to the majority of other lipid classes—partition into the upper, aqueous phase. From there, they can be isolated by a solid phase extraction and separated from neutral GSLs by anion exchange chromatography [107], such as with DEAE (=diethylaminoethyl) Sephadex [108]. 

*O*-Acetylation of sialic acids and also ganglioside lactonization [90] are modifications that are lost under alkaline conditions. These are often applied to remove glycerophospholipids that contain fatty acids in ester linkage [109]. If information on these modifications is desired, gangliosides from tissues can be determined without alkaline treatment, for example, after chloroform/methanol extraction in a ratio of 1 : 2 and a subsequent partition step [110].

Separation of gangliosides according to their glycan composition is achieved by thin layer chromatography (TLC) [111] and by HPLC and other techniques that can be coupled to mass spectrometry [112]. This facilitates their identification by mass spectrometry and is required for their characterization by staining with suitable antibodies [113, 114], lectins, or other binding proteins [115]. Although not required for mass-spectrometric profiling, separated ganglioside classes can also be further separated according to their ceramide structure by reversed phase chromatography [116–118].

Quantification can be achieved by staining and densitometry, or—if suitable standard substances are available—by mass spectrometry. Since the biosynthetic machinery generates heterogeneities within both, the lipid and the glycan part, comprehensive ganglioside analysis is a highly demanding task within lipidomics [101]. In addition to glycoforms that are also well known from glycoprotein analysis, “lipoforms” [119] become increasingly important to understand ganglioside metabolism and function. Various protocols for ganglioside determination by mass spectrometry have been developed [101]. They are largely based on electrospray mass spectrometry as ionization technique; but also MALDI plays a role. The available methods range from preanalytics to bioinformatic data handling and include imaging methods using MALDI and Secondary Ion mass spectrometry (SIMS) to determine the spatial distribution of the analytes [101]. In addition to their constitution, little is known about the conformation of gangliosides in their native, membrane-bound surroundings. X-ray data are not available for gangliosides, although isolated glycans have been investigated by various means. For a simulation of GM3 conformations in a bilayer, compare [120], which, for example, shows that the glucose moiety of GM3 is buried within phosphatidylcholine head groups. 

## 5. Biosynthesis

The diversity of cell surface glycans, including that of gangliosides, is generated within the Golgi apparatus [121], and the heterogeneities within the ceramide part result from the biosynthesis of ceramide at the endoplasmic reticulum (ER). *De novo* synthesis of gangliosides can be distinguished from salvage processes [122, 123], in which sialic acids, sugars, fatty acids, and sphingoid bases are recycled. The latter process can predominate by far in differentiated cells. 

### 5.1. Ceramide Biosynthesis

**Figure 9:**
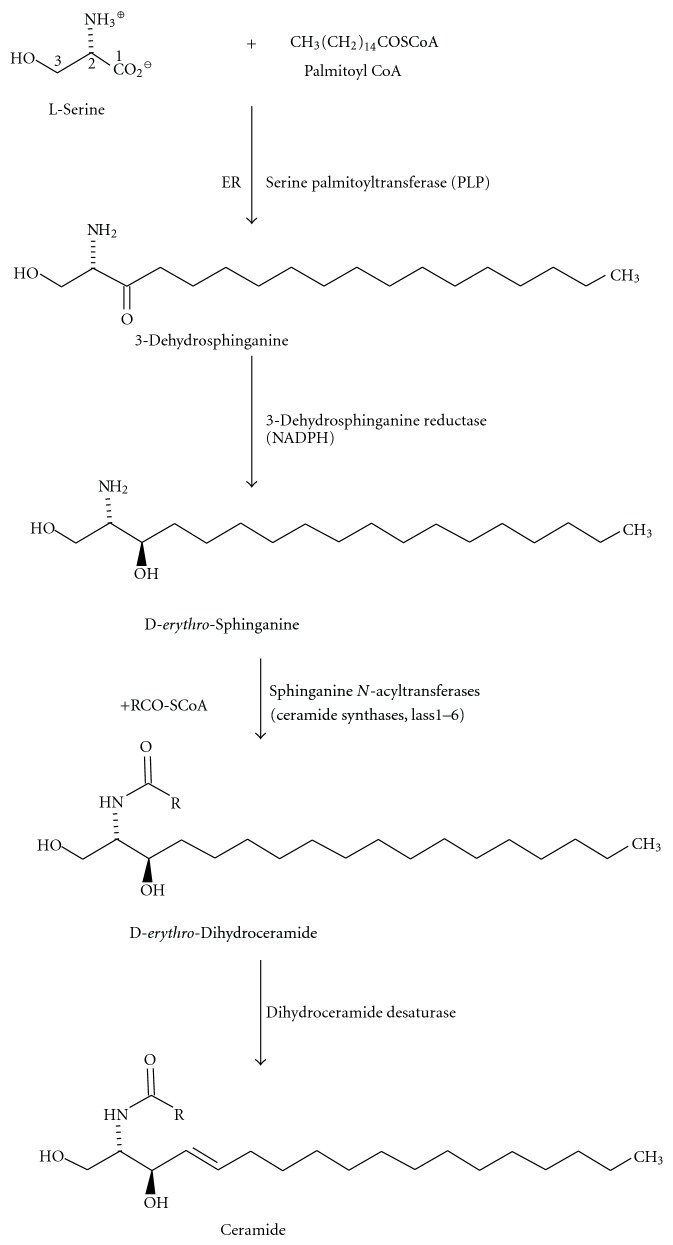
Ceramide biosynthesis, shown for a ceramide with C_18_ sphingosine. R = alkyl; chain length depend on the availability of the corresponding acyl CoAs and the identity of the ceramide synthase.

Ganglioside biosynthesis starts with the formation of ceramide (Figure 9) at the cytoplasmic leaflet of the ER membrane [124–126]. The first step, the condensation of L-serine and a coenzyme A-activated fatty acid is catalyzed by the pyridoxal phosphate-dependent serine palmitoyltransferase (SPT) [127]. The incorporation of L-serine into GSLs can be used to monitor their *de novo* biosynthesis using L-serine radiolabelled in the position 3 (the carbon in position 1 is lost as carbon dioxide). In the brain, the external supply of L-serine by astrocytes is essential for neuronal lipid biosynthesis and brain development [128]. In agreement with this observation, genetically engineered rodents with deficient phosphoglycerate dehydrogenase required for L-serine formation from D-glucose show drastically reduced ganglioside levels, defects in brain morphogenesis, and drastically reduced lifespan [129, 130].

The next step in sphingolipid biosynthesis is the NADPH-dependent reduction of 3-ketosphinganine to sphinganine by 3-ketosphinganine reductase, followed by acylation of sphinganine to dihydroceramides of different chain lengths [131]. During salvage, also other sphingoid bases are acylated by *N*-acyltransferases of the lass family. Lass 1 encodes ceramide synthase 1, which is expressed in the brain and involved in the formation of the membrane anchor of gangliosides. In mice, spontaneous recessive mutations in the lass1 gene are associated with cerebellar ataxia and Purkinje cell degeneration [132]. Although the ceramide part of brain gangliosides contains mostly nonhydroxylated fatty acids, apparently all members of the lass family are also able to transfer the corresponding 2-hydroxy-fatty acids [133]. Dihydroceramides are dehydrogenated to ceramide by the dihydroceramide desaturase des1 [134], or hydroxylated to phytoceramides by des2. Ceramide is the common precursor of GSLs and sphingomyelin and is transported to the Golgi apparatus at least in part in a protein-dependent manner by the transport protein CERT [135–137]. 

### 5.2. General Aspects of Ganglioside Formation

GSL synthesis continues by the stepwise transfer of nucleotide-activated monosaccharide units first on ceramide and then on GSLs with growing glycan chains. Glycosidation is coupled to exocytosis through the Golgi apparatus to the plasma membrane [138] at the rate of bulk vesicle flow [139].

The complex ganglioside and GSL glycoforms on eukaryotic cell surfaces are generated by only a few enzymes that act within a combinatorial biosynthetic pathway [140, 141]. The first glycosyltransferases involved in ganglioside biosynthesis have been characterized in the laboratories of Roseman and Basu [142]. According to the number of sialic acids connected to the “inner” galactosyl residue, ganglio-series gangliosides are classified into members of the 0-, a-, b-, and c-series (Figure 12). b-Series gangliosides contain the Neu5Ac*α*2,8Neu5Ac sequence, which is commonly not found in glycoproteins. Higher members of these different subseries can be formed by the action of the same glycosyltransferases, which show less specificity than those acting early in the pathway [143–147]. 

**Figure 10:**
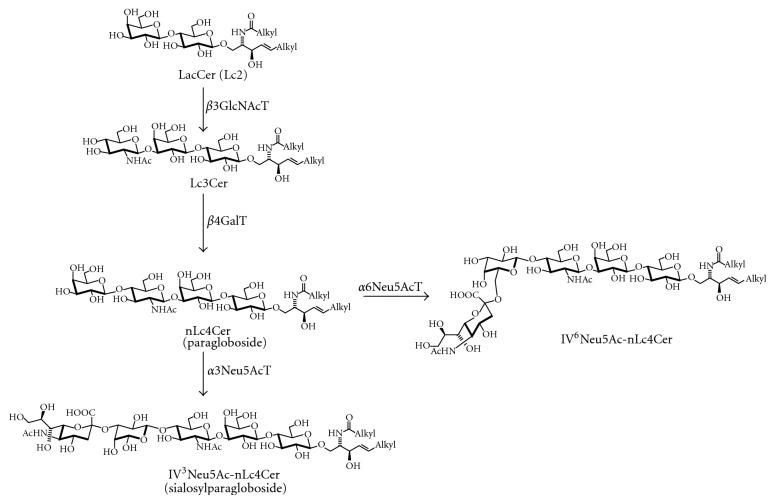
Reaction of *β*1,3-*N*-acetyl-glucosaminyltransferase (*β*3GlcNAcT) and examples of downstream gangliosides of the neolacto series.

The glycosyltransferases and sialyltransferases [148, 149] of the ganglioside biosynthetic pathway are expressed in a cell-type- and developmental-dependent fashion. Ganglioside pattern changes during the development of the brain [150], and after differentiation, differences in glycolipid composition have even been found between different neuronal cell types [151]. In addition, ganglioside patterns vary between different cell types and change with the differentiation of the cell. As an example, *β*1,3-*N*-acetylglucosaminyltransferase expression, which leads to the formation of glycolipids of the lacto and neolacto series (Figure 10), is high during murine embryonic development and decreases after birth to undetectable levels in most cell types [152, 153]. In adult animals, expression is high in spleen [154], and in cerebellum, it is restricted to Purkinje cells [155].

GSLs including gangliosides are formed biosynthetically at intracellular membranes from which they are transported to the plasma membrane by exocytotic membrane flow [138]. While many human diseases are known that are due to defects in GSL and sphingolipid degradation, the only known human disease caused by a defect glycosyltransferase of ganglioside biosynthesis is the human autosomal recessive infantile-onset symptomatic epilepsy syndrome, which is caused by a nonsense mutation in the gene encoding GM3 synthase [156].

A principal difference between ganglioside biosynthesis in the Golgi apparatus and degradation in the endolysosomal compartment is that during GSL formation, membrane-bound glycosyltransferases interact with their membrane-bound glycolipid substrates by diffusion within the two-dimensional plane of the lipid bilayer. Therefore, reaction rates can become independent of the reaction volume and obey two-dimensional enzyme kinetics. This means that kinetic constants can be normalized on lipid surface area instead of reaction volume, for example, in terms of the amount of membrane protein [157]. As a consequence, glycosyltransferases that lack their transmembrane domain lose most of their activity towards membrane bound substrates [158]. During degradation in endosomes and lysosomes, the glycosidases are soluble enzymes, and the substrates are membrane-bound. This explains in part the requirement for endosomal and lysosomal lipid-transfer proteins for the degradation of GSLs with short glycan chains, which is not the case in biosynthesis. In addition to ganglioside biosynthesis in the Golgi apparatus, there are also indications ganglioside formation by plasma membrane-associated glycosyltransferases [159].

### 5.3. Gala Series

In the monoglycosylceramides glucosylceramide (GlcCer) and galactosylceramide (GalCer), which are also called cerebrosides, the hexosyl residues are present in *β*-anomeric configuration. GalCers with *α*-configuration occur only in lower organisms [42] and are highly immunogenic for mammals [160]. Most gangliosides are biosynthetically derived from GlcCer; only ganglioside GM4 is derived from GalCer. Ganglioside GM4 has been discovered as a minor component of human brain gangliosides [161], where it is localized within myelin [162]. It also occurs, for example, on erythrocytes, kidney, and in the intestine and is abundant in some fish species. However, the most frequently found members of the gala series are GalCer and sulfatide (GalCer-3-sulfate) in oligodendrocytes, Schwann cells, kidney, testis, and intestine. They are present in high concentrations in the multilamellar layers of the myelin where they are required for glial adhesion [163], apparently via interaction between the carbohydrate head groups of sulfatide and GalCer on different myelin layers [164]. Myelin lipids contain the highest fraction of 2-hydroxy-fatty acids, which are formed by fatty acid hydroxylase-2 [165]. Their presence in gala-series GSLs contributes to carbohydrate-carbohydrate interactions between the GSLs [166]. 

In contrast to GalCer synthase, GlcCer synthase appears to be dispensable for oligodendrocytes [167]. While ceramide galactosylation catalyzed by UDP-glucose:ceramide galactosyltransferase (GalT3) [168] occurs at the ER membrane, the later steps of gala-series GSL biosynthesis, formation of sulfatide [169], digalactosylceramide [170], and ganglioside GM4 take place in the lumen of the Golgi apparatus. In contrast to most glycosyltransferases in ganglioside biosynthesis, which are type II transmembrane proteins, ceramide galactosyltransferase is a type I transmembrane protein with the catalytic domain on the luminal side of the ER [171]. According to data obtained in zebrafish and mice, GM4 can be formed by ST3Gal V, which can also make GM3. Therefore, GM4 and GM3 formation appear to depend on the availability of their precursors, GalCer and LacCer [172]. Little is known about the function of GM4. It can interact with the myelin basic protein, shows immunosuppressive properties, and can prevent experimental allergic encephalomyelitis in guinea pigs [173].

### 5.4. Gangliosides Derived from Glucosylceramide

The first step in the biosynthesis of most gangliosides is the transfer of a glucose residue from UDP glucose to ceramide catalyzed by UDP-glucose:ceramide glucosyltransferase [174, 175]. Although GlcCer and GalCer synthases catalyze similar reactions, their cDNAs share no sequence homology. Ceramide glucosyltransferase is a type III transmembrane protein. It forms noncovalent dimers or oligomers [176] with their C-terminal catalytic domains in the cytosol [177]. Since the formation of GlcCer occurs on the cytoplasmic face [178] and that of LacCer on the luminal site of the Golgi membrane [179], glucosylceramide has to be translocated across a membrane. This is mediated by a flippase of unknown identity: the ABC-transporters, ABC-B1 and -C1, translocate short chain GlcCer analogs through the Golgi membrane [180, 181]. Transversal translocation can be carried out after transport of GlcCer by the cytoplasmic lipid-transfer protein FAPP2 (four-phosphate adaptor protein 2) either to the ER [182], where it might be translocated by an uncharacterized flippase [183], or at the trans-Golgi [184]. A part of the GlcCer pool can reach the cytosolic leaflet of the plasma membrane where it can be degraded by the *β*-glucosidase Gba2 [185]. Candidate cytosolic GlcCer-transporters are the glycolipid transfer protein GLTP and FAPP2.

**Figure 11:**
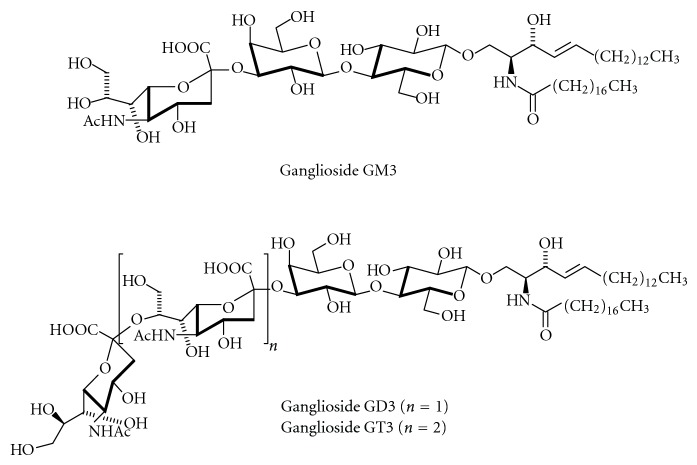
Structures of hematosides.

The biosynthesis of higher gangliosides occurs on the luminal face of the Golgi apparatus [186], so that their glycan chains are orientated extracytoplasmic. LacCer is formed by galactosyltransferase I, which transfers a galactose residue from UDP galactose to glucosylceramide [187]. Further carbohydrate residues are transferred in a stepwise manner to the growing glycan chains. LacCer and its sialylated derivatives, the hematosides GM3, GD3, and GT3 (Figure 11) serve as precursors for complex gangliosides of the 0-, a-, b-, and c-series. 

**Figure 12:**
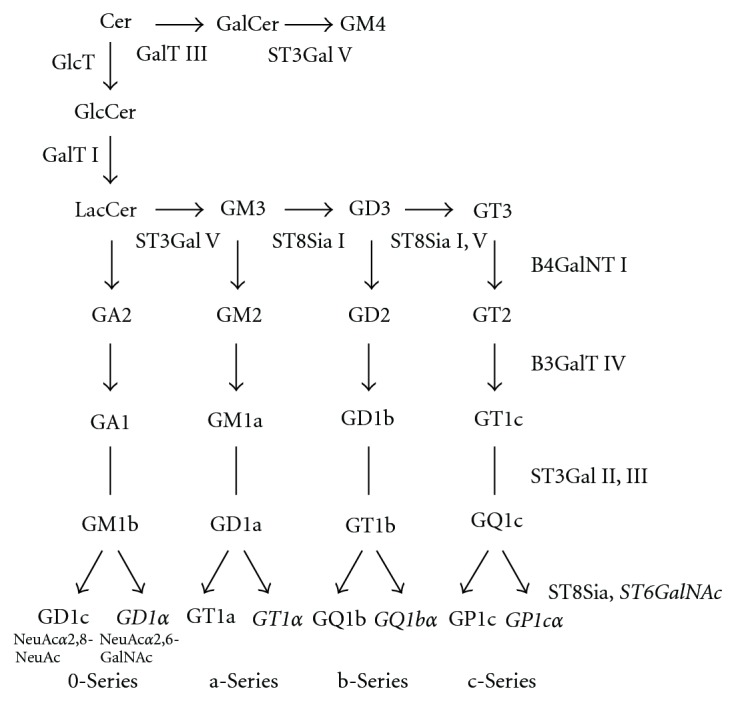
Ganglioside biosynthesis (modified from [141]).

**Figure 13:**
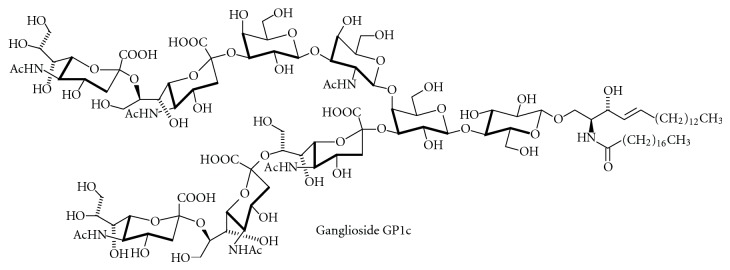
Structure of a c-series ganglioside.

These different series (Figure 12) are characterized by the presence of no (0-series), one (a-series), two (b-series) or three sialic acids residues linked to the position 3 of the “inner” galactosyl residue. In adult mammalian brain, gangliosides from the 0- and c-series are found only in trace amounts, and GM1b and GD1*α* are transiently expressed during chick brain biogenesis [188]. 0-series gangliosides (GM1b, GD1c, and GD1*α*) are found in genetically engineered mice deficient in ST3Gal V (GM3 synthase), where they are present in amounts that correspond to the total ganglioside content of normal animals [189]. These mice are not able to form GM3 and higher gangliosides of the a–c-series. They display altered glucose homeostasis with an accelerated insulin receptor signalling pathway, a key finding that demonstrates the inhibition of the insulin receptor by GM3 or a higher ganglioside derived from it *in vivo* [189].

c-Series gangliosides (Figures 12 and 13) are formed during mammalian brain development where they are thought to be involved in growth, differentiation, and migration of neuronal cells. They are abundant in fish brain, and in adult rats; they occur in liver, kidney, and pancreas [190] and in tumors such as glioma. The transferases that catalyze the first steps in ganglioside biosynthesis show high specificity towards their glycolipid substrates. The relative amounts of LacCer, GM3, GD3, and GT3 seem to determine the amount of 0-, a-, b-, and c-series gangliosides. The glycosyltransferases that act late in this pathway represent a kind of assembly line and transfer the respective carbohydrates to glycosyl acceptors that differ only in the number of sialic acid residues bound to the “inner” galactose residue. The complex “*α*-”gangliosides with sialic acid moieties in *α*2,6-glycosidic linkage to *N*-acetylgalactosamine residues is specific for cholinergic neurons [191] and has been added later to the biosynthetic scheme [192]. In mice, the sialyltransferases that form gangliosides GD1a and GT1b have been identified as ST3Gal II and ST3Gal III [193].

### 5.5. Genetically Engineered Mice

A significant advance towards understanding the function of the complex ganglioside pattern found in eukaryotic cells is the development of mice with defects in distinct biosynthetic steps [194]. A mouse melanoma cell line deficient in GlcCer and GlcCer-derived GSLs was viable and showed only minor changes in cellular morphology and growth rate. From these observations it was concluded that GSLs including gangliosides are not essential for animal survival [195]. Later, it was reported that mice with targeted disruption of the ceramide glucosyltransferase gene displayed no cellular differentiation beyond the primitive germ layers and died around day 7.5 of embryonic development [62]. Mice deficient in B4GalNT I (GM2-synthase) are not able to form GM2, GD2, and higher gangliosides derived from them. Although these animals show only subtle impairment of brain function [196], they exhibit multiple defects, such as axonal degeneration, defects in myelination [197] and motor function [198], or an impaired response of T cells to interleukin 2 [199], only to mention a few. Later studies showed that CD4- and CD8-positive T cells require different ganglioside subsets for activation [200]. The mutant male mice are sterile and also show morphological and functional defects in the testis [196]. Further examination of GalNAc-transferase deficient mice revealed that GM1-deficiency is accompanied by Parkinson-like symptoms, which could be rescued by L-Dopa or the membrane permeable GM1-analog Liga20 (see Figure 19) [201]. This is in agreement with a series of reports that GM1 can alleviate symptoms in models of Parkinson disease, for example, [202]. In Parkinson disease, anionic lipids and especially GM1 inhibit aggregation of *α*-synuclein to cytotoxic fibrils [203].

Mice deficient in ST8Sia I (GD3-synthase) do not form GD3 and b-series gangliosides. They have a normal life span and are without detectable developmental defects [204]. When these mice were crossbred with mice carrying a disrupted B4GalNT I gene, the resulting double mutant mice express only ganglioside GM3 as their major ganglioside. These “GM3-only-mice” are extremely susceptible to sound stimuli, develop lethal seizures, and display a sudden death phenotype [204]. Double knockout mouse deficient in B4GalNT I and ST3Gal V (GM3-synthase) are not able to form any ganglioside of the ganglio-series. These animals are severely diseased and show elevated levels of LacCer, LacCer-sulfate, and traces of other gangliosides that are present also in normal brain [205].

### 5.6. Regulation

Sphingolipid biosynthesis is a highly regulated process and also coordinated with sterol and glycerolipid biosynthesis. Sphingolipids are major regulators of lipid metabolism and activate sterol-regulatory element binding proteins (SREBPs) [206]. The sphingomyelin synthase-related synthase, the ceramide transporter CERT, and proteins of the orosomucoid- (Orm)-familie seem to play key roles in sphingolipid homeostasis [207]. Ganglioside pattern are characteristic for a cell type in a certain differentiation state, and, for example, mice deficient in GM3-synthase that cannot form the typical brain gangliosides show a ganglioside content similar to that of normal animals [189]. How exactly the relative amounts of gangliosides are controlled is not clear [208], but the transcriptional regulation of transferase genes seems to be a key point [208]. The picture gets more complicated by the fact that different transferase isoforms with different properties can be present: three murine GM3-synthase isoforms that arise from two transcripts have been characterized. One is resident in the ER membrane, the two others in the Golgi, but with different half-life [209]. In addition, the kinetic parameters of the transferases, their topological organization within the Golgi apparatus, or spatial neighborhood to other transferases will influence the resulting ganglioside pattern. An attempt has been made to calculate glycolipid pattern on the bases of the kinetic constants of the transferases, that were estimated from the steady state concentrations of the glycolipid substrates in intact cells [210]. Contradictory results have been reported on the subcellular localization of the glycosyltransferases involved in the biosynthesis of ganglio-series gangliosides [211]. An additional feature of ganglioside biosynthesis and its regulation [212] is the formation of functional complexes, as predicted by Roseman [213]. In these complexes [140], the glycosyltransferases do not only form functional platforms, but can also show altered activity and suborganellar localization [214]. One of these complexes characterized in certain CHO cells [215] comprises B4GalNT I and B3GalT IV (Figure 12), so that it can accept GM3 and release GM1. This might explain why the brain contains large amounts of GM1 and GD1a, but little GM2. Also GalT I, ST3Gal V, and ST8Sia I can form such a complex [216].

## 6. Degradation

### 6.1. General

The constitutive degradation of gangliosides takes place in endosomes and lysosomes. In addition, also the plasma membrane-associated sialidase Neu3 [217, 218] can degrade gangliosides and is, for example, highly expressed on melanoma cells [219]. Even the nuclear envelope contains sialidases, with Neu3 in the inner and Neu1 in the outer nuclear membrane [220]. Lysosomal ganglioside degradation takes place after the endocytosis of parts of the plasma membrane at intraendosomal and intralysosomal membranes and related lipid aggregates. This requires the presence of suitable glycosidases [221], of an appropriate pH, in some cases also of lipid-transfer proteins, and of an appropriate composition of the ganglioside-containing membranes [222].

**Figure 14:**
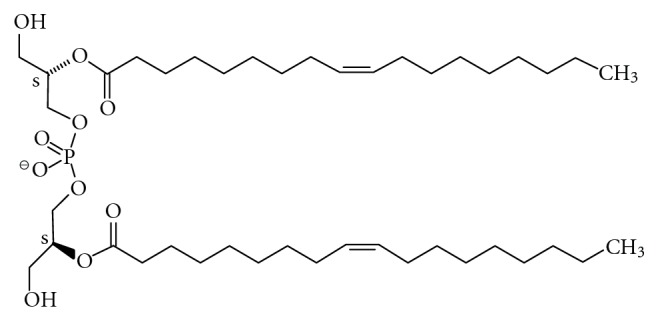
Bis(monoacylglycero)phosphate with two oleic acid moieties.

**Figure 15:**
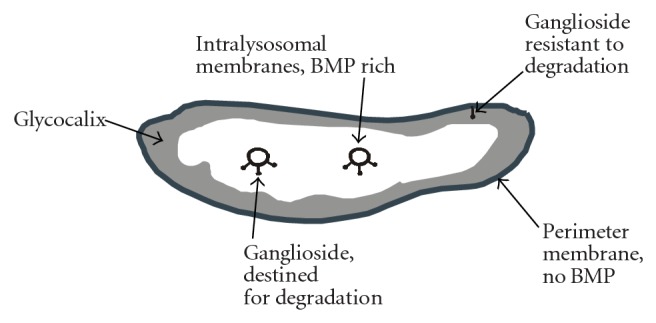
Lysosomal membrane pools.

As proposed in 1992, two different membrane pools are present in endosomes and lysosomes [223] (Figure 15). They differ in lipid- and protein composition and function. While the luminal membrane pool that is derived from the plasma membrane or by autophagy is degraded, the perimeter membrane (Figure 15) is protected from degradation by various means [224]. This ensures the integrity of the compartment, which can be abolished during apoptosis [224]. A marker lipid that is exclusively found in luminal membranes [225] is bis(monoacylglycero)phosphate (BMP; Figure 14), chemically incorrect also named as lysobisphosphatidic acid (LBPA). BMP plays a key role for membrane degradation [226] and is formed from phosphatidylglycerol [227]. Due to its *sn*1, *sn*1′ configuration, it is only slowly degraded by lipases and persists on inner membranes, in which it can amount up to 70% of total phospholipids [228]. With a predicted p*K*_*A*_ value of about 2, BMP is negatively charged even at lysosomal pH. *In vitro* studies show that negatively charged lipids are required for binding of lysosomal proteins to membranes. Although other negatively charged lipids such as dolichol phosphate or phosphatidylinositol can be present on luminal membranes, BMP appears to be the key factor that distinguishes this membrane pool from the perimeter membrane. On the other hand, the perimeter membrane of endosomes and lysosomes shows an entirely different lipid and protein composition. It is protected by a glycocalyx formed by highly *N-*glycosylated integral membrane proteins [229, 230], and ganglioside GM3 present in this membrane is resistant to degradation [231].

**Figure 16:**
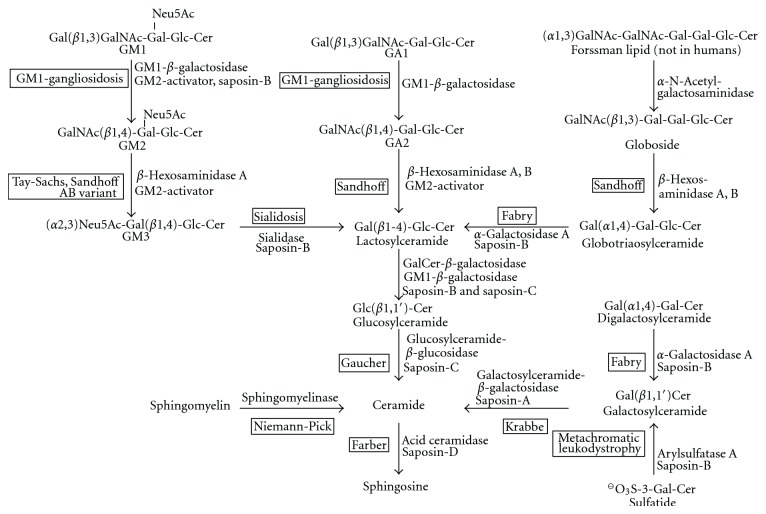
Lysosomal ganglioside degradation pathway (modified from [233]). Names of inherited diseases (in boxes), enzymes, and lipid-transfer proteins required *in vivo* are given.

Ganglioside degradation starts with the action of glycosidases that cleave off monosaccharide units from the non-reducing end of the ganglioside glycan chains. This happens in a sequential manner, which explains the different human diseases that are associated with defects in this pathway. The glycosidases are soluble enzymes in the lumen of endosomes and lysosomes. It turned out that their activity is not sufficient towards GSL substrates with cleavage sites in proximity to the intralysosomal membrane surface. Although also other factors play a role, this can be attributed to steric hindrance by adjacent membrane components that impede the access of the soluble enzyme. For example, in wild-type and GM2-activator deficient fibroblasts, radiolabelled GD1aGalNAc (Figure 3), which has the same terminal trisaccharide as GM2, is degraded in the absence of the GM2-activator protein, while the degradation of GM2 itself is strictly dependent on the presence of the activator [232]. As glycosidase substrates, GSLs with four carbohydrate residues or less require the additional presence of small lipid binding glycoproteins, either the GM2 activator protein or one of the four saposins A–D. These act in part as lipid-transfer proteins that extract the membrane-bound substrates and present them to the hydrolases. They have different specificities and mechanisms of action [233]. In the case of gangliosides, at least the GM2-activator protein and saposin-B participate in the degradation of GM1, GM2, and GM3 (Figure 16). 

*In vitro*, in addition to enzymes and activator proteins, also an appropriate membrane-lipid composition of the ganglioside-containing membrane is required for degradation [222]. Saposin-A [234] and saposin-B [235] extract membrane lipids much better from membranes that are rich in BMP and poor in cholesterol. BMP also increases the ability of the GM2 activator to solubilize lipids [236] and stimulates the hydrolysis of membrane-bound GM1 by GM1 *β*-galactosidase [237] and of ganglioside GM2 by *β*-hexosaminidase A [236]. BMP also stimulates hydrolysis of the kidney sulfatide with ganglio-series GSL-core SM2 (gangliotriaosylceramide-II^3^ sulfate) by *β*-hexosaminidases A and S in the presence of the GM2 activator [238, 239]. Cholesterol, which is known to stabilize lipid bilayers, has to be transported from intraendosomal membranes to the NPC1 protein resident in the endosomal perimeter membrane by the soluble lipid-transfer protein NPC2. *In vitro*, this transfer is greatly stimulated by BMP and strongly inhibited by sphingomyelin [240]. 

### 6.2. Selected Key Proteins for Ganglioside Degradation

#### 6.2.1. Saposin-B

Saposins are small, water-soluble lysosomal lipid-binding and -transfer proteins of about 8–11 kDa molecular weight. They are derived from a common precursor protein, prosaposin, by proteolytic processing. Saposins belong to a family of proteins with conserved three-dimensional fold [241] and occur as homo- and heterodimers and -oligomers. The first saposin has been characterized in 1964 as the so-called sulfatide activator since it enables the degradation of sulfatide by arylsulfatase A [242]. Today this protein is known as saposin-B or sap-B. Saposin-B has many functions: it is a lipid-binding protein with broad specificity [243] and forms water-soluble lipid-protein complexes [244]. With respect to gangliosides, it is able to stimulate the degradation of ganglioside GM1 by GM1-*β*-galactosidase [237]. Studies in cultured human skin fibroblast derived from saposin-B and prosaposin-deficient patients show that it is also required for the degradation of GM3 [245, 246]. It is important to note that glycosylation of saposin-B is essential for some of its functions and that human patients without this postranslational modification die, although the unglycosylated variant protein is present in lysosomes [235].

Mechanistically, saposin-B dimers seem to act similar to the GM2 activator: X-ray data indicate that they can adopt two conformations, an open one and a closed one [247]. According to a model view, the open conformation interacts directly with the membrane and extracts the lipid ligand. This is accompanied by a change to the closed conformation in which the ligand is exposed to the degrading enzyme in a water-soluble activator-lipid complex. Human patients with an inherited deficiency of saposin-B develop an atypical form of metachromatic leukodystrophy with the accumulation of sulfatides, digalactosylceramide, and globotriaosylceramide [248] (see Figure 16 for structures). Saposin-B knockout mice show enhanced levels of sulfatides especially in brain and kidney [249]. 

#### 6.2.2. The GM2 Activator

The GM2 activator is a small glycoprotein of 17.6 kDa in its deglycosylated form and is required for the degradation of ganglioside GM2 by *β*-hexosaminidase A *in vivo* [250]. Inherited deficiency of the GM2-activator protein leads to the AB variant of GM2 gangliosidoses [251]. Based on the X-ray structure [252, 253] and data from photoaffinity labeling [254], in some respects the GM2-activator acts in a way similar to saposin-B. A more detailed picture of the binding mode was derived from binding studies using a spin-labelled GM2 activator to phosphatidylcholine bilayers [255]. The protein can extract a variety of lipids, which has been exploited for assay development [256]. However, its major function is to form a water-soluble GM2-protein complex that is the native Michaelis-Menten substrate of *β*-hexosaminidase A [250]. Negatively charged lipids such as BMP, dolichol phosphate, or phosphatidylinositol increase the extraction efficiency towards GM2 [236], GM1 [237], and other lipids [257] from liposomal membranes. Binding characteristics of the GM2 activator are altered by the presence of a His tag [257].

In Langmuir experiments, the GM2-activator protein is able to penetrate into a phospholipid monolayer, but only when the lateral pressure is below a critical value, which depend on the lipid composition and is in the range from 15 to 25 mN/m [258]. In addition to its function as a ganglioside-transfer protein, the GM2 activator binds also other lipids like phosphatidylcholine [259] and platelet activating factor (PAF) and inhibits its action [260, 261]. It is not clear whether the GM2 activator displays inherent hydrolytic activity towards lipid substrates such as platelet activating factor [262] or phosphatidylcholine [259]. Apparently unrelated to its GM2 transfer property is the function of the GM2 activator as adipokine [263]. GM2-activator orthologs might serve different functions in other organisms, for example as a pheromone-binding protein in *Drosophila* [264], or an inhibitor of PAF-induced chemotaxis in nematodes [265].

The saposins and the GM2-activator play major roles in the transfer of lipid antigens to membrane-resident CD1-proteins [266, 267]. 

#### 6.2.3. Proteins Required for GM1 Degradation

GM1-*β*-galactosidase is a protein of 64 kDa, which is derived from an 88-kDa precursor [268, 269]. An alternatively spliced, enzymatically inactive *β*-galactosidase form of 67 kDa is an elastin/laminin-binding protein [270]. GM1-*β*-galactosidase is part of a lysosomal multienzyme complex, together with the so-called protective protein (carboxypeptidase A), sialidase, and *N*-acetylaminogalactose-6-sulfate sulfatase [271]. GM1-*β*-galactosidase catalyzes the hydrolytic cleavage of several *β*-galactosides. The hydrolysis of ganglioside GM1 to GM2 requires the presence of either the GM2-activator protein, or saposin-B [56], or, *in vitro*, of an appropriate detergent.

#### 6.2.4. Proteins Required for GM2 Degradation

GM2 is degraded by the cleavage of the *N*-acetylgalactosaminyl residue by *β*-hexosaminidases. In mice, the substrate specificity of the murine lysosomal sialidase allows for a significant cleavage also of the sialic moiety in GM2 (to yield GA2) [272]. Cleavage of the GalNAc residue requires the presence of the GM2-activator protein *in vivo*, or of an appropriate detergent *in vitro*. Three gene products participate in GM2 hydrolysis, the *β*-hexosaminidase *α*- and *β*-chains, and the GM2-activator protein. *β*-Hexosaminidases are dimers that result from the combination of their *α*- and *β*-subunits and differ in properties such as stability and substrate specificity. *β*-Hexosaminidase A with subunit composition *α*,*β* cleaves terminal *β*-glycosidically linked *N*-acetyl-glucosamine and *N*-acetylgalactosamine residues from negatively charged and uncharged glycoconjugates by a retaining double-displacement mechanism. The enzyme has two active sites, one on the *α*-chain and the other on the *β*-chain [273]. *β*-Hexosaminidase B (*ββ*) [274, 275] predominantly cleaves uncharged substrates such as GA2 and oligosaccharides with terminal *N*-acetyl-hexosamine residues (see also Figure 16). *β*-Hexosaminidase S (*αα*) is thermolabile and of secondary significance for GM2 degradation, but it contributes to the degradation of glycosaminoglycans and sulfated glycolipids [238].

## 7. Pathobiochemistry 

Defects in enzymes and other proteins required for lysosomal degradation of complex lipids and of oligomeric or polymeric biomolecules lead to inherited diseases, the lysosomal storage diseases [276]. They can be classified according to the stored substances, as sphingolipidoses [277], mucopolysaccharidoses, mucolipidoses, glycoprotein-, and glycogen-storage diseases [278, 279]. Ganglioside degradation is impaired in the gangliosidoses and secondarily also in other sphingolipid storage diseases [280]. The principles [281] governing pathogenesis [282, 283] and therapy of sphingolipidoses [284] are also valid for the ganglioside storage diseases. 

Key factors are the residual activity of the degrading system, which determines the course of the disease [285, 286], and the cell-type-specific expression of storage material. Due to the cell-type-specific expression of gangliosides, the central nervous system is especially affected in the gangliosidoses. In sphingolipidoses in general, the storage lipids coprecipitate other hydrophobic substances present in the endolysosomal compartment, lipids and proteins, as secondary storage products [280]. In Niemann-Pick disease, type C, which is a primary defect of endosomal cholesterol transport, a secondary accumulation of sphingomyelin (therefore the name Niemann-Pick) and of gangliosides is observed that is also of therapeutic relevance [287, 288]; for a remarkable treatment of Niemann Pick C1 fibroblasts with a histone deacetylase inhibitor, compare [289]. Secondary storage of gangliosides GM2 and GM3 occurs also in Hurler disease [290] (mucopolysaccharidosis type I; *α*-L-iduronidase deficiency). Lipid storage produces a kind of traffic jam [291, 292], which interferes with lipid transport and lysosomal function. Primary and secondary storages substances can impair nutrient delivery via the endolysosomal system: as demonstrated in mouse models of GM1 gangliosidoses and in a variant form of the GM2 gangliosidoses, Sandhoff disease, iron homeostasis is impaired in the animals, and supplementation of the animals with iron ions increased their life expectancy by nearly 40% [293]. Since also autophagy can be impaired in lysosomal storage diseases [294], both pathways may lead to a shortage of nutrients.

Ganglioside degradation is impaired in the gangliosidoses. In another disease, galactosialidosis, the primary defect of carboxypeptidase A (protective protein), leads to a secondary loss of *β*-galactosidase and sialidase Neu1 accompanied by GM1 storage [271]. Gangliosidoses are caused by defects in the genes encoding glycosidases or lipid-transfer proteins that are required for lysosomal ganglioside degradation. The theoretical basis for the therapeutic approaches towards gangliosidoses is the “threshold theory” [286], which predicts that the ratio of substrate influx into the lysosomes and the degradation capacity determine the course of the diseases. Both parameters can be addressed by different therapeutic approaches [281].

### 7.1. GM1 Gangliosidosis

GM1 gangliosidosis is caused by an inherited deficiency of GM1-*β*-galactosidase (acid *β*-galactosidase; GLB1; EC3.2.1.23) [295]. After the description of the first patients [296] it became also known as Landing diseases [297]. It is a rare disease with an autosomal recessive mode of inheritance and characterized by the accumulation of GM1 and GA1 (Figure 16) in neuronal cells [12]. According to the substrate specificity of the variant enzyme in the patients, an inherited defect of the *β*-galactosidase can also lead to another disease, Morquio disease, type B. Three clinical forms of GM1 gangliosidosis can be distinguished, infantile (type 1) GM1 gangliosidosis with the developmental arrest and progressive deterioration of the nervous system in early infancy and a life expectancy of about 2 years, late infantile/juvenile form (type 2), and an adult/chronic form (type 3). Dysmorphic changes characteristic for Morquio disease type B are less prominent or completely absent in these clinical forms.

In addition to GM1, other enzyme substrates accumulate, such as GA1 (Figure 16) [12], oligosaccharides from glycoproteins, and intermediates of keratin sulfate degradation [268]. These substances are stored in different organs, according to their major site of biosynthesis. Lysosomal GM1 accumulation in neurons leads to the degeneration of the nervous system. Like in other storage diseases, an inflammatory response [298], neurorestorative properties of excess ganglioside GM1 [299] in the plasma membrane, and an unfolded protein response [300] contribute to pathogenesis. Such as in other sphingolipidoses [281], severity and progression of the disease correlate with the residual enzymatic activity in cells and body fluids.

Morquio type B disease clinically resembles a mild phenotype of Morquio A disease, where keratan sulfate accumulates due to *N*-acetyl-galactosamine-6-sulfatase deficiency. Like GM1 gangliosidosis, Morquio type B is due to the inherited defect of GM1-*β*-galactosidase. It is characterized by the predominant storage of keratan sulfate and oligosaccharides with terminal *β*-galactosyl residues. Patients show generalized skeletal dysplasia without involvement of the nervous system and without hepatosplenomegaly; for a clinical description, compare [268]. Differences between GM1 gangliosidosis and Morquio B disease can be attributed to a lower affinity and activity of *β*-galactosidase variants towards substrates with Gal-*β*1,4-GlcNAc motifs in Morquio patients compared to the Gal-*β*1,3-GalNAc motive present in ganglioside GM1 [301]. There is no causal therapy available for GM1-gangliosidosis; however, progress is made towards the development of pharmacological chaperones also for this lysosomal disease [302–304].

### 7.2. GM2-Gangliosidoses

The GM2-Gangliosidoses are caused by defects in degradation of ganglioside GM2 [305]. The three variant forms of the GM2-gangliosidoses are named according to the hexosaminidase isoenzyme that remains intact. The B-variant, in its infantile course better known as Tay-Sachs disease, is caused by the deficiency of hexosaminidases A and S, but with normal hexosaminidase B. The 0 variant, or Sandhoff disease, is caused by the deficiency of the *β*-chain and the resulting deficient activity of *β*-hexosaminidases A and B (therefore, none of the major enzymes is intact), however with the remaining activity of *β*-hexosaminidase S. The AB-variant—*β*-hexosaminidases A and B (and S) intact—results from mutations in the GM2-activator gene; so that tissue samples from the patients are able to degrade GM2 in detergent-containing enzyme assays. 

#### 7.2.1. Tay-Sachs Disease

Clinically, the B variant of GM2 gangliosidoses can be subclassified into infantile, juvenile, chronic, and adult onset forms. The infantile form, Tay-Sachs disease, has a higher prevalence among Ashkenazi Jews with a heterozygote frequency of 1 : 27. Affected children are normal at birth and show first symptoms, such as mild motor weakness, a cherry red spot in the central retina, and increased startle reaction between 3 and 6 months of life. Progressive deterioration with weakness, hypotonia, or poor head control leads to a vegetative state and death often between the second and fourth year of life. Juvenile and adult course is observed in patients with a higher residual activity of the variant hexosaminidase A [285]. Symptoms are very heterogeneous; for a clinical description, compare [305].

**Figure 17:**
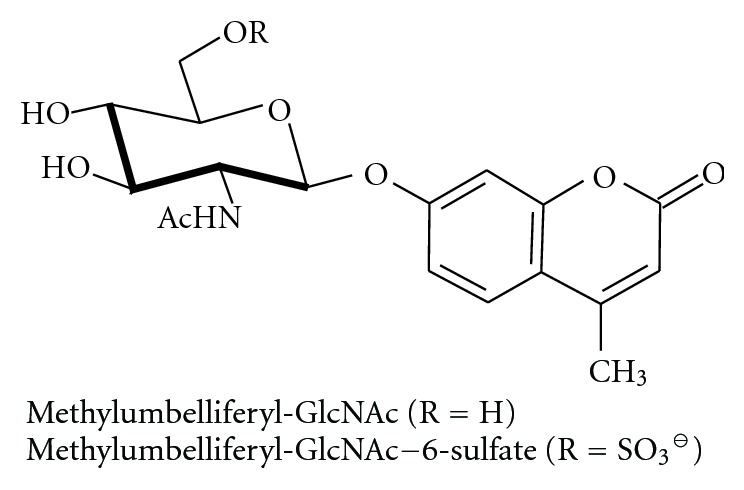
Synthetic substrates for the determination of *β*-hexosaminidase A (R = SO_3_^−^) and *β*-hexosaminidases A + B (R = H).

The B1 variant of GM2 gangliosidoses [309, 310] was very difficult to elucidate: synthetic uncharged substrates used for diagnosis such as MufGlcNAc (Figure 17; for kinetic parameters see [311]) were cleaved, suggesting the presence of *β*-hexosaminidase, and also the GM2 activator was present. As it turned out, the B1 variant differs enzymatically from the B variant by an altered substrate specificity of the variant *β*-hexosaminidase A. While uncharged substrates are cleaved, no activity is detected towards GM2 and towards sulfated, negatively charged [312] synthetic fluorogenic substrates. In the B1 variant, the function of the *α*-chain active site is defective, but subunit association, enzyme processing, and the activity of the *β*-chain are not impaired. Homozygous patients with the B1 mutation show the course of the juvenile disease; compound heterozygotes with a B1 and a null allele show a late infantile course.

#### 7.2.2. Sandhoff Disease

The 0 variant of GM2 gangliosidosis was the first gangliosidosis for which the underlying enzymatic defect was identified [14]. Due to the deficiency of two enzyme activities, *β*-hexosaminidases A and B, storage of negatively charged glycolipids characteristic for Tay-Sachs disease and, in addition, of uncharged substrates such as GA2 in the brain and globoside in visceral organs (Figure 16) is observed. In infantile Sandhoff disease, patients show clinical and pathological manifestations of Tay-Sachs disease (infantile B variant) and in addition also organomegaly and slight bone deformations. For further symptoms and the description of juvenile and adult forms, compare [305].

#### 7.2.3. AB Variant of GM2 Gangliosidosis

The AB variant is due to the deficiency of the GM2-activator protein [251], with intact *β*-hexosaminidases A and B (and S), therefore the name. The disease is characterized by accumulation of GM2 and GA2 (for structures, see Figure 16). The clinical picture [305] resembles that of Tay-Sachs disease with a delayed appearance of symptoms; an animal model is available [313].

#### 7.2.4. Pathogenesis and Therapy

**Figure 18:**
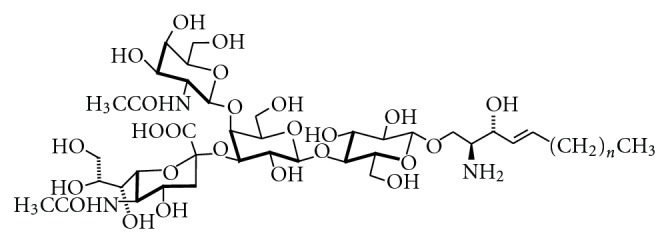
LysoGM2 (*n* = 12, 14).

Although lysosomal GM2 as the major storage compound in GM2 gangliosidoses is neither toxic nor immunogenic, its accumulation induces inflammatory responses as demonstrated for glycoconjugates in the murine model of Sandhoff disease [108]. Huge axon hillock enlargements, the so-called meganeurites, have been observed in neurons of patients with different lysosomal storage diseases, which might be attributed to the storage substance GM2 and contribute to synaptic dysfunction [314]. As in other sphingolipidoses [281], the corresponding (more toxic) lysolipid, in this case lysoGM2 (Figure 18), is elevated [315, 316] and contributes to the pathogenesis. LysoGM2 has been suggested as a biomarker for Tay-Sachs and Sandhoff disease [317]; for occurrence and role of lysoGSLs in acquired diseases, compare [318]. Despite naturally occurring animal models of GM2 gangliosidoses in dogs, cats, and pig, murine models are used for therapy studies. Since the mouse model of Tay-Sachs disease is largely asymptomatic, the mouse model of Sandhoff disease is used for most studies [272].

Despite some success in the experimental treatment of juvenile and adult patients as well as in the animal models, there is no causal therapy available for the severe forms of the GM2 gangliosidoses. The limitations of the substrate reduction approach, which reduces the GM2 influx into the lysosomal compartment, have been evaluated by a genetic experiment: Sandhoff-disease mice were crossbred with mice defective in GM2 synthase. The lifespan of these animals was much longer than that of Sandhoff-disease mice, but instead of GM2 storage they developed a oligosaccharide storage, neurological disease [319]. 

Therapeutic approaches such as bone-marrow transplantation [320], enzyme-replacement therapy with recombinant highly phosphomannosylated *β*-hexosaminidase A [321], or transplantation of neural stem cells [322] have been investigated in the animal model of the 0 variant, substrate-reduction therapy with *N*-butyl deoxynojirimycin [323, 324], and with pyrimethamine as pharmacological chaperone [325, 326] in adult patients and gene therapy in endothelial cells [327]. Treatment of the accompanying inflammation is beneficial [328].

### 7.3. Selected Other Aspects

In addition to inherited diseases, ganglioside levels can also be altered in several acquired diseases [318]. For example, gangliosides play roles in neurological diseases such as Alzheimer's [329], Parkinson, or Huntington's disease [330]. In cancer, ganglioside expression can also be altered in tumor cells with an impact on signalling and tumor-host interactions [331]. [Neu5Gc]GM3 [332], GD2, GD3, GM2, and fucosylGM1 are regarded as tumor-associated antigens [333] and are targets for the immunotherapy of cancer [334]. 

Also several neuropathies including variant forms of Guillain-Barré and Miller-Fisher syndrome are caused by serum antibodies against gangliosides [335].

There are only a few therapeutic roles for gangliosides, especially since they can induce neuropathies. In the past, gangliosides isolated from bovine brain have been investigated and also applied to human patients to improve neural repair and for the treatment of stroke [336, 337]. Also the direct application of ganglioside GM1 into the brain of patients with Alzheimer disease has been evaluated [338]. As indirect roles, the inhibition of ganglioside biosynthesis for the treatment of insulin resistance [339], the interference with microbial binding to gangliosides [340], or the reduction of neurotoxicity with Liga20 [341] has to be mentioned. 

## 8. Functional Aspects

A plethora of functions has been attributed to gangliosides [342], for example, for GM3 [343], the most abundant ganglioside in most mammalian cell types, but not in neurons, or for GM1 [344]. In general, gangliosides mediate their function via interaction with soluble or membrane-bound binding molecules outside the cell (“trans” interaction), or by influencing properties of proteins within the same membrane (“cis” interaction) [32, 345–348]. “Trans” interactions occur between the glycan part of gangliosides on the one side with lectins on the other side. Also gangliosides contribute to the chemical high-density sugar code of cell surfaces [349]. For example, GM1 can be recognized by galectin-1 [350] and sialic acids in *α*2,3-linkage are recognized by the sialic acid binding immunoglobulin lectins Siglec-4, *α*2,6-sialosides by Siglec-2, and *α*2,8-sialosides by Siglec-7 [347]. Also carbohydrate-carbohydrate interactions can play a role [351, 352]. Although interactions between individual carbohydrate residues [353] are weak, clustering of gangliosides offer the possibility for multivalent interactions, if they are not buried under glycoprotein glycans. Apparently, GM3 on mouse melanoma B16 cells can mediate cell adhesion to mouse lymphoma L5178 cells by binding to GA2 (gangliotriaosylceramide, GalNAc*β*1,4Gal*β*1,4Glc*β*1,1Cer; see Figure 12 or Figure 16) [354]. Within the nervous system, gangliosides act in a “trans” manner with the myelin-associated glycoprotein MAG. MAG recognizes NeuAc*α*2-3Gal*β*1-3GalNAc-termini on axonal gangliosides, an interaction that is essential for axon–myelin stability and axon regeneration [355]. 

“Cis” interactions can happen via a direct interaction, or, indirectly, via the properties of the membrane or of putative-membrane domains [356]. This way gangliosides influence the activities of receptor-tyrosine kinases in the plasma membrane, such as the receptors of epidermal-growth factor, nerve growth factor, and insulin and therefore cell signaling [357]. For example, GM1 enhances TrkA neurotrophin receptor-activation in a “cis”-manner [358]; for a brief overview of proteins affected by certain gangliosides, compare, for example, [359]. Since the characterization of lipid microdomains in living cells is difficult, some conclusions can be drawn from *in vitro* experiments. For example, GM3 inhibits the autophosphorylation of purified EGFR reconstituted into the proteoliposomes of defined lipid compositions, but not the EGF binding [360]. There are indications that gangliosides may not act only in an autonomous manner, but might also support the formation of distinct membrane phases, although it is a matter of debate to which extent this operates *in vivo*. It is believed that gangliosides are not homogeneously distributed on the cell surface, but segregate into membrane domains together with GPI-anchored proteins, sphingomyelin and cholesterol. Such rafts have been supposed to be the physiological surroundings of many membrane proteins, although no rigid proof for their existence has been provided. Ganglioside plays a largely unexplored role for membrane structure [356]. Due to their large hydrated head groups, they stabilize membrane areas with positive curvature [361]. A multitude of reports propose a segregation of gangliosides and other GSLs with cholesterol and GPI-anchored proteins into the lipid platforms known as “rafts” in the membranes of living cells [362–365]. Since most of the applied methods (detergent extraction, antibody, and toxin staining) constitute a bias towards the formation of such domains [366], the existence, size, and lifetime of rafts are a matter of debate. From thermodynamic considerations, it is clear that procedures such as detergent extraction can (or have to) produce artificial results and are not suitable for raft characterization [367–370]. Although it has been demonstrated that the treatment of cellular membranes with detergents causes the redistribution of gangliosides and GPI-anchored proteins [371, 372], these techniques are still applied and not always critically examined. Experiments in living cells using STED microscopy [308] and other visualization techniques [373] point to an upper limit of lifetime and size of such domains in the range of 20 ms and 20 nm.

In addition, sialic acids and oligosialic acids present on gangliosides can modulate membrane surface charge density, the pH at the membrane surface, and membrane potentials [374]. In planar lipid bilayers, ganglioside GD1a can increase the excitability of voltage-dependent sodium channels [375]. 

### 8.1. Infection

“Cis” and “trans” interactions of gangliosides play multiple roles in the immune system [376] and in infectious diseases [377, 378], where gangliosides act as cellular receptors and coreceptors for viruses, bacteria, and microbial toxins. The most prominent example is GM1 as the receptor for cholera toxin [379]; other examples are the toxin of *Clostridium botulinum* and the SabA adhesin of *Helicobacter pylori* [380] that bind to cell surface gangliosides of the host [381]. Binding of sialylated cell surface glycoconjugates to Siglecs [382] on white blood cells is used within innate and adaptive immune responses to distinguish between self and nonself and to dampen autoimmune responses [383]. Many pathogens use sialic acids on cell surface glycoconjugates for cellular entry, for example, periodontal pathogens [384]. Recent examples include ganglioside GT1b, which seems to be the host cell receptor for the Merkel cell polyomavirus [385]. This virus has been identified as the cause of Merkel cell carcinoma, an aggressive type of skin cancer. Also sialidase-insensitive rotaviruses recognize sialic acid, for example, on ganglioside GM1, which is not substrate of all sialidases due to its branched structure [386] and the glycan present in ganglioside GD1a serves as host receptor for the adenoviruses that cause epidemic keratoconjunctivitis [387].

## 9. Tools

### 9.1. Loss of Function

Gangliosides are secondary gene products. Their function can be analyzed by knockout experiments, where in cells, tissues, or organisms their formation or degradation is interrupted by genomic, posttranscriptional, or chemical strategies. Especially valuable were genetically engineered mice with defects in ganglioside biosynthesis [388], which revealed, for example, a role of gangliosides in calcium homeostasis [389], neural repair [390], or neurological diseases [330]. Also investigations in human patients [277], genetically engineered mammals [391], and other organisms [392] allowed insight into various aspects of ganglioside metabolism and transport. Also mutant and silenced cells have been applied for functional studies. *In vitro* systems such as liposomes or planar monolayers allow investigations that are to difficult to be carried out in cells. For example, when GM3 is incorporated into liposomes, a phase separation into GM3-rich and GM3-poor phases occurs above a certain GM3 content [393]. This would fit to reports on GM3-enriched microdomains in living cells. 

In experimental approaches, ganglioside biosynthesis can be modulated by inhibitors [394]. Also the enhancement of ganglioside biosynthesis can be used, for example, chemically, or by the introduction of glycosyltransferase encoding cDNA in cultured cells [395, 396]. An enantiomer of the glucosylceramide synthase inhibitor D-*threo*-PDMP (PDMP = 1-phenyl-2-decanoylamino-3-morpholino-1-propanol), L-*threo*-PDMP, acts as an enhancer of ganglioside biosynthesis by upregulating glycosyltransferases. This was accompanied by increased neurite outgrowth [397]. Additional possibilities are the generation of mutant cells, for example for GM2 synthase [398] or by posttranscriptional silencing like RNA interference. While even complex systems like cultured cells can survive without GSLs, they are required for the development of multicellular organisms [399]. 

### 9.2. Structurally Defined and Modified Gangliosides

Structurally homogenous gangliosides and ganglioside probes that are modified by isotopes, fluorescence, chemical reporter groups, photoaffinity, or affinity ligands are valuable tools for the analysis of ganglioside function, metabolism, and transport. These tools are available by total or partial chemical synthesis, or by biosynthetic incorporation of suitable—for example, photolabile—*N*-acylmannosamine precursors into gangliosides [400] using the methodology for biosynthetic sialic acid modification developed by Kayser et al. [401]. For enzymological, transport, and crosslinking studies, tritium and ^14^C are incorporated into different positions of gangliosides, but also radioiodination is possible in the presence of aryl residues [402, 403].

Ganglioside total synthesis is a time-consuming and demanding task and usually performed only in specialized laboratories for examples, see [49, 50]. It relies predominantly on the sequential glycosidation of a 3-*O*-protected azidosphingosine [404] with suitably protected and activated glycosyl donors. This includes the trichloroacetimidates [405] as well as methods for *α*-selective sialylation reactions [406]. Also chemoenzymatic procedures have been developed, where different glycosidation steps are catalyzed by glycosyltransferases [407] or glycosidases [408], or where oligosaccharyl fluorides are coupled to native or fluorescent ceramide anchors using an engineered endoglycoceramidase (glycosynthase) [49, 409]. Ganglioside oligosaccharides, such as those of GM3, GM2, GM1, GD3, and GT3, can be produced by genetically engineered bacterial strains. For the biotechnological production of ganglioside head groups, lactose can be internalized in *E. coli* as a precursor to be used as acceptor for glycosyltransferases [410–415]. An application of the ganglioside biosynthetic machinery is the preparative production of neoglycolipids with ganglioside head groups using a lung squamous-cell carcinoma line (RERF-LC-A) and 12-azidododecyl *β*-lactoside as a suitable primer [416].

GSLs isolated from natural sources can be used for the preparation of chemically modified derivatives [417] like labelled GSLs [418, 419], or those of enhanced metabolic stability [420]. The chemical release of the ganglioside glycan chain can be achieved by osmium tetroxide/periodate treatment of protected gangliosides [421, 422], or by ozonolysis of native gangliosides [423]. Initially, both methods give rise to ganglioside aldehydes, which are not isolated but subsequently fragmented by alkaline treatment, or, if desired, are isolated for further applications [424]. The glycan part can also be released enzymatically by ceramide glycanase [425, 426]. Lysogangliosides that lack the acyl moiety at the sphingosine nitrogen can be prepared by chemical procedures [427, 428], or by enzymatic treatment of gangliosides with sphingolipid *N*-deacylase [429–431].

**Figure 19:**
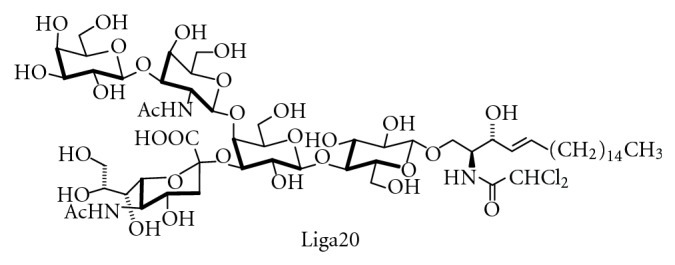
Structure of Liga20, a semitruncated and halogenated GM1 analog that can pass the blood-brain barrier.

Lysogangliosides (see Figure 18) can be used for the introduction of fluorescence, spin, or radiolabels or other modifications into the lipid backbone of gangliosides. A semitruncated, dihalogenated GM1 analog that is able to pass the blood-brain barrier is Liga20 [398] (Figure 19). There are also several approaches to the synthesis of photoactivatable GSL derivatives [432]. 

It has to be noted that when gangliosides are added to the medium of cultured cells, they are largely present as oligomers in the form of micelles or vesicles, as monomers bound to proteins, and as free monomers. In aqueous surroundings, gangliosides form aggregates of different size and shape [361, 433]. In most cases these are micellar structures of 280–630 kDa [361], in the case of the gangliosides with small head groups, GM3 and GM4 also vesicles [361, 434, 435]. The critical micellar concentrations of GSLs are in the range of 10^−9^–10^−5 ^M [436] and depend on temperature, pH, and, in part, on the method of determination. Typical values are 3.4 × 10^−9^ M for GM3 to 3.9 × 10^−8^ M for GT1b [434].

**Figure 20:**
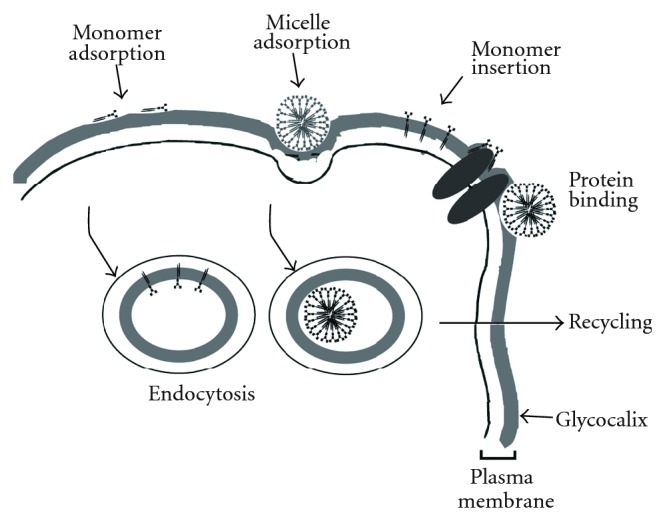
Schematic ganglioside uptake by cultured cells (modified from [306]).

**Figure 21:**
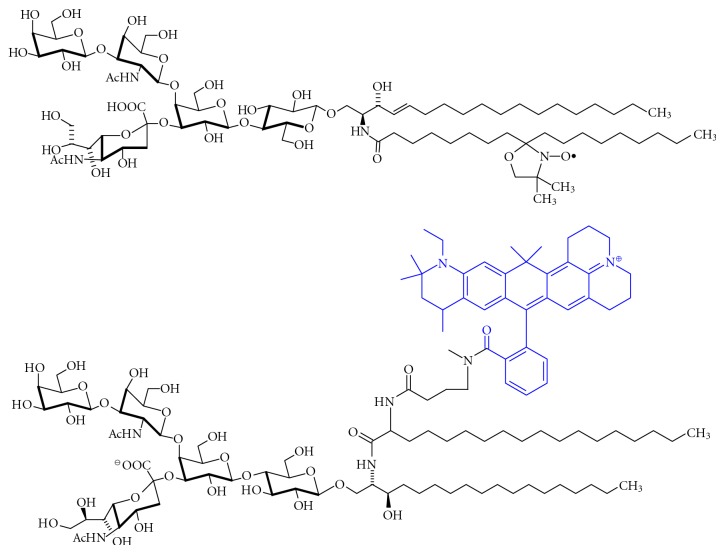
Example of a GM1 analog [307] spin labelled with a 4,4-dimethyl-oxazolidine-1-oxyl- (DOXYL-) residue and a fluorescent GM1 analog used for STED microscopy [308].

Uptake of exogenously added gangliosides by cells in culture [437] can proceed in different ways [306, 420] (Figure 20). With the aid of radiolabelled [438] and spin-labelled gangliosides [307] (Figure 21), three modes of adherence have been distinguished: 60–90% of the exogenous ganglioside consist in loosely associated micelles and also monomers, which can be removed by delipidated serum proteins. A second fraction is attached to cellular proteins in a trypsin-labile fashion, and, finally, a trypsin-stable fraction is presumably inserted into the plasma membrane of the cell. Only the last fraction is in the topologically correct, native orientation. When bound to proteins, the offrate of gangliosides with native alkyl chain lengths can be very low. This is not the case for synthetic, semitruncated derivatives of higher solubility, which are frequently used, but show different intracellular transport behaviour compared to native gangliosides [420]. Fluorescently labelled glycosphingolipids [439] have been applied, but also their properties can differ significantly from the ones of native glycosphingolipids.

Gangliosides can also be transferred from cultured donor to acceptor cells that are separated by a membrane [440]. By this process known as “shedding” [441], tumor cells can release up to 0.5% of their gangliosides per hour [442].

Fluorescent ganglioside probes that bear the fluorophore in at the membrane-water interphase (Figure 21) behave physicochemically more like native gangliosides. Such compounds have been used as probes for STED microscopy [308] or to quantify the transfer capacity of GM2 activator in a liposomal FRET assay system [443]. 

## 10. Conclusion

Despite the fast development of analytical and biophysical tools, the analytical determination of ganglioside pattern, their spatial resolution, and their correlation with function is still a challenge. Especially a convincing characterization of ganglioside-containing membrane domains in living cells and of their roles at the cellular level would constitute a considerable advance in the field.

## Figures and Tables

**Table 1:** GSL series.

Series	Core structure
Arthro	GlcNAc*β*1,3Man*β*1,4Glc*β*1,1′Cer
Gala	Gal*α*1,4Gal*β*1,1′Cer
Neogala	Gal*β*1,6Gal*β*1,6Gal*β*1,1′Cer
Ganglio	Gal*β*1,3GalNAc*β*1,4Gal*β*1,4Glc*β*1,1′Cer
Globo	GalNAc*β*1,3Gal*α*1,4Gal*β*1,4Glc*β*1,1′Cer
Isoglobo	GalNAc*β*1,3Gal*α*1,3Gal*β*1,4Glc*β*1,1′Cer
Lacto	Gal*β*1,3GlcNAc*β*1,3Gal*β*1,4Glc*β*1,1′Cer
Neolacto	Gal*β*1,4GlcNAc*β*1,3Gal*β*1,4Glc*β*1,1′Cer
Muco	Gal*β*1,3Gal*β*1,4Gal*β*1,4Glc*β*1,1′Cer
Mollu	Fuc*α*1,4GlcNAc*β*1,2Man*α*1,3Man*β*1,4Glc*β*1,1′Cer
Schisto	GalNAc*β*1,4Glc*β*1,1′Cer
Spirometo	Gal*β*1,4Glc*β*1,3Gal*β*1,1′Cer

## Abbreviations

Cer: Ceramide, N-Acylsphingosine
GlcCer: Glc*β*1Cer
GalCer: Gal*β*1Cer
Sulfatide: Sulfate3Gal*β*1Cer (I^3^-sulfate, GalCer)
LacCer: Gal*β*1,4Glc*β*1Cer
GbOse3Cer: Gal*α*1,4Gal*β*1,4Glc*β*1Cer (Gb_3_Cer)
GbOse4Cer: GalNAc*β*1,3Gal*α*1,4Gal*β*1,4Glc*β*1Cer (Gb_4_Cer)
GA2: GalNAc*β*1,4Gal*β*1,4Glc*β*1Cer (Gg_3_Cer)
GA1: Gal*β*1,3GalNAc*β*1,4Gal*β*1,4Glc*β*1Cer (Gg_4_Cer)
GM1b: Neu5Ac*α*2,3Gal*β*1,3GalNAc*β*1,4Gal*β*1,4Glc*β*1Cer (IV^3^Neu5AcGg_4_Cer)
GD1c: Neu5Ac*α*2,8Neu5Ac*α*2,3Gal*β*1,3GalNAc*β*1,4Gal*β*1,4Glc*β*1Cer (IV^3^(Neu5Ac)_2_Gg_4_Cer)
GD1*α*: Neu5Ac*α*2,3Gal*β*1,3(Neu5Ac*α*2,6)GalNAc*β*1,4Gal*β*1,4Glc*β*1Cer (IV^3^Neu5AcIII^6^Neu5AcGg_4_Cer)
GM4: Neu5Ac*α*2-3Gal*β*1Cer (I^3^Neu5Ac*α*GalCer)
GM3: Neu5Ac*α*2,3Gal*β*1,4Glc*β*1Cer (II^3^Neu5AcLacCer)
GM2: GalNAc*β*1,4(Neu5Ac*α*2,3)Gal*β*1,4Glc*β*1Cer (II^3^Neu5AcGg_3_Cer)
GM1a: Gal*β*1,3GalNAc*β*1,4(Neu5Ac*α*2,3)Gal*β*1,4Glc*β*1Cer (II^3^Neu5AcGg_4_Cer)
GD1a: Neu5Ac*α*2,3Gal*β*1,3GalNAc*β*1,4(Neu5Ac*α*2,3)Gal*β*1,4Glc*β*1Cer (IV^3^Neu5AcII^3^Neu5AcGg_4_Cer)
GT1a: Neu5Ac*α*2,8Neu5Ac*α*2,3Gal*β*1,3GalNAc*β*1,4(Neu5Ac*α*2,3)Gal*β*1,4Glc*β*1Cer (V^3^(Neu5Ac)_2_II^3^Neu5AcGg_4_Cer)
GT1*α*: Neu5Ac*α*2,3Gal*β*1,3(Neu5Ac*α*2,6)GalNAc*β*1,4(Neu5Ac*α*2,3)Gal*β*1,4Glc*β*1Cer (IV^3^Neu5AcIII^6^(Neu5Ac)_2_Gg_4_Cer)
GD3: Neu5Ac*α*2,8Neu5Ac*α*2,3Gal*β*1,4Glc*β*1Cer (II^3^(Neu5Ac)_2_LacCer)
GD2: GalNAc*β*1,4(Neu5Ac*α*2,8Neu5Ac*α*2,3)Gal*β*1,4Glc*β*1Cer (II^3^(Neu5Ac)_2_Gg_3_Cer)
GD1b: Gal*β*1,3GalNAc*β*1,4(Neu5Ac*α*2,8Neu5Ac*α*2,3)Gal*β*1,4Glc*β*1Cer (II^3^(Neu5Ac)_2_Gg_4_Cer)
GT1b: Neu5Ac*α*2,3Gal*β*1,3GalNAc*β*1,4(Neu5Ac*α*2,8Neu5Ac*α*2,3)Gal*β*1,4Glc*β*1Cer (IV^3^Neu5AcII^3^(Neu5Ac)_2_Gg_4_Cer)
GQ1b: Neu5Ac*α*2,8Neu5Ac*α*2,3Gal*β*1,3GalNAc*β*1,4(Neu5Ac*α*2,8Neu5Ac*α*2,3)Gal*β*1,4Glc*β*1Cer (IV^3^(Neu5Ac)_2_II^3^(Neu5Ac)_2_Gg_4_Cer)
GQ1b*α*: Neu5Ac*α*2,3Gal*β*1,3(Neu5Ac*α*2,6)GalNAc*β*1,4(Neu5Ac*α*2,8Neu5Ac*α*2,3)Gal*β*1,4Glc*β*1Cer (IV^3^(Neu5Ac)_2_III^6^(Neu5Ac)_2_Gg_4_Cer)
GT3: Neu5Ac*α*2,8Neu5Ac*α*2,8Neu5Ac*α*2,3Gal*β*1,4Glc*β*1Cer (II^3^(NeuAc)_3_LacCer)
GT2: GalNAc*β*1,4(Neu5Ac*α*2,8Neu5Ac*α*2,8Neu5Ac*α*2,3)Gal*β*1,4Glc*β*1Cer (II^3^(NeuAc)-Gg_3_Cer)
GT1c: Gal*β*1,3GalNAc*β*1,4(Neu5Ac*α*2,8Neu5Ac*α*2,8Neu5Ac*α*2,3)Gal*β*1,4Glc*β*1Cer (II^3^(Neu5Ac)_3_Gg_4_Cer)
GQ1c: Neu5Ac*α*2,3Gal*β*1,3GalNAc*β*1,4(Neu5Ac*α*2,8Neu5Ac*α*2,8Neu5Ac*α*2,3)Gal*β*1,4Glc*β*1Cer (IV^3^Neu5AcII^3^(Neu5Ac)_3_Gg_4_Cer)
GP1c: Neu5Ac*α*2,8Neu5Ac*α*2,3Gal*β*1,3GalNAc*β*1,4(Neu5Ac*α*2,8Neu5Ac*α*2,8Neu5Ac*α*2,3)Gal*β*1,4Glc*β*1Cer (IV^3^(Neu5Ac)_2_II^3^(Neu5Ac)_3_Gg_4_Cer)
GP1c*α*: Neu5Ac*α*2,3Gal*β*1,3(Neu5Ac*α*2,6)GalNAc*β*1,4(Neu5Ac*α*2,8Neu5Ac*α*2,8Neu5Ac*α*2,3)Gal*β*1,4Glc*β*1Cer (IV^3^Neu5AcIII^6^Neu5Ac,II^3^(Neu5Ac)_3_Gg_4_Cer)
GD1b-lactone: II^3^[Neu5Ac-(2–8,1–9)-Neu5Ac]Gg_4_Cer
GalNAc-GD1a: GalNAc*β*1,4Gal*β*1,3GalNAc*β*1,4(Neu5Ac*α*2,3)Gal*β*1,4Glc*β*1Cer (IV^3^Neu5AcII^3^Neu5AcGg_5_Cer)
Neu5Ac: N-Acetyl-neuraminic acid
Glc: Glucose
Gal: Galactose
GalNAc: N-Acetyl-galactosamine.
